# Language confidence and job satisfaction among foreign-born nurses in Japan: mediating effect of workplace discrimination and moderating effect of immigration duration

**DOI:** 10.1186/s12912-024-02116-3

**Published:** 2024-07-02

**Authors:** Jing Hua, Akiko Kondo, Congcong Wang, Sambuu Ganchulun

**Affiliations:** 1 International Nursing Development, Graduate School of Health Care Sciences, Tokyo Medical and Dental University, 1-5-45 Yushima Bunkyo-Ku, Tokyo, 113-8519 Japan; 2 Mongolia-Japan Hospital of Mongolian National University of Medical Sciences, Ulaanbaatar, Mongolia

**Keywords:** Non-native nurses, Workplace discrimination, Job satisfaction, Language confidence

## Abstract

**Aim:**

This study explored the relationship between language confidence and job satisfaction, the mediating role of workplace discrimination, and the moderating role of immigration duration among foreign-born nurses in Japan.

**Introduction:**

Job satisfaction is an important factor in preventing migrant nurses’ turnover intentions; however, the relationships among language confidence, immigration duration, workplace discrimination, and job satisfaction among foreign-born nurses remain unclear.

**Methods:**

A cross-sectional study was conducted. Data were collected between June and August 2022 through an online survey of nurses who were born outside of Japan but were currently working as registered nurses in Japan. PROCESS v4.0 Macro for SPSS 28.0 was applied to analyze the effect of language confidence on job satisfaction, the mediator effect of workplace discrimination (model 4), and the moderator effect of immigration duration (model 15).

**Results:**

Data from 187 participants were analyzed. The results showed that 1) foreign-born nurses’ language confidence was negatively correlated with workplace discrimination and positively correlated with job satisfaction; 2) workplace discrimination played a partially mediating role between language confidence and job satisfaction; and 3) immigration duration positively moderated the relationship between language confidence and job satisfaction.

**Conclusion:**

Foreign-born nurses with stronger confidence in their proficiency in Japanese perceived less workplace discrimination and higher job satisfaction. Workplace discrimination acted as a mediator in the relationship between language confidence and job satisfaction, and this relationship was strengthened with longer migration periods. Managers and policymakers should implement policies and strategies to combat workplace discrimination and provide tailored support to improve foreign-born nurses’ job satisfaction, which may contribute to their retention in Japan.

**Supplementary Information:**

The online version contains supplementary material available at 10.1186/s12912-024-02116-3.

## Introduction

Migration of nurses is a global phenomenon. Foreign-born nurses increased from 11 to 16% between 2000 and 2016 across the countries of the Organization for Economic Cooperation and Development (OECD) [1]. The main emigration movement was from low-income to high-income countries; nursing shortages in receiving countries and poor work environments in the countries of origin pushed this movement [2, 3]. Japan also faces a serious nursing shortage due to its growing aging population [4]. Japan has flourished in receiving migrant nurses since 2008 under the Economic Partnership Agreement, and the number of foreign healthcare workers has increased by approximately 4.6 times from 2012 to 2022 [5]. Therefore, a considerable number of foreign-born nurses are expected to enter the Japanese workforce in the near future.

Despite foreign-born nurses entering the workforce in receiving countries, high turnover rates have contributed to a growing nursing shortage [2]. The Japanese Ministry of Health, Labour and Welfare [6] reported that the turnover of foreign workers was twice as high as that of Japanese workers. Moreover, the International Council of Nurses (ICN) encourages receiving countries to develop suitable strategies to retain foreign-born nurses and establish a multicultural, sustainable workforce [2].

Job satisfaction is an indicator of social well-being and contributes to preventing non-native nurses’ turnover intention [7]. According to Balante et al. [8] and Viken et al. [9], migrant nurses frequently experience language barriers and workplace discrimination that may affect job satisfaction and patient safety. However, most previous studies were qualitative and described migrant nurses’ lived and workplace experiences, including being denied advancement opportunities, having an unequal workload and low confidence in communicating with patients and colleagues [10, 11]. Lack of confidence in a second language is a major cause of language obstacles for immigrants who need to learn a second language to live and work in receiving countries [12].

The Job Demands-Resources (JD-R) Theory states that the balance between job demands and job resources influences an employee's job satisfaction (social well-being) [13]. Job demands are aspects of the job that require continuous emotional or cognitive effort. Previous studies have shown workplace discrimination as a job demand that increases the workload and depletes employees' energy, leading to reduced employees’ well-being [14, 15]. Job resources are those that assist in achieving work goals, reducing job demands, and stimulating personal development. Language confidence can be a personal job resource that facilitates effective communication and decreases misconceptions in the workplace [16]. Previous quantitative studies have explored the relationship between organizational commitment and support, nationality, and non-native nurses’ job satisfaction [17, 18], but limited results have explored the impact of language confidence (job resources) and workplace discrimination (job demands) on migrant nurses’ social well-being (job satisfaction) in Japan. Prior studies indicated that foreign-educated nurses with lower perceived discrimination in the workplace showed higher job satisfaction [17, 19]. However, the relationship between language confidence and workplace discrimination or job satisfaction remains unclear. Moreover, Bakker and Demerouti [20] suggest that job resources can reduce the negative effects of job demands on employee outcomes, demonstrating the mediating role of job demands. Therefore, workplace discrimination may play a mediating role in the relationship between language confidence and job satisfaction.

Immigration duration refers to the length of time an immigrant has lived in the receiving country. In the early period of migration, workplace discrimination is an important obstacle that arises among migrant workers [11], and their perceived discrimination has decreased over time [21]. Immigrants gradually become familiar with the receiving country’s cultural norms and workplace expectations [22]. Additionally, their second language competence and confidence are also improved [12], which might potentially help them navigate the effects of discrimination in the workplace. A recent study conducted in Japan indicated that foreign-educated nurses who worked longer showed higher job satisfaction [19]. According to the JD-R theory, when migrant nurses stay longer in the receiving country and become more integrated, these job demands (workplace discrimination) might be reduced. This reduction might buffer the negative effects of discrimination on job satisfaction. Similarly, a longer immigration period generally leads to better cultural integration [22], which might enhance the effectiveness of language confidence as a job resource, amplifying the positive effects of language confidence on job satisfaction. Furthermore, a previous study has shown that immigration duration plays a positive moderating role in the relationship between perceived discrimination and self-esteem among the migrant population [23]. Thus, this study posits that immigration duration may moderate the relationship between workplace discrimination, language confidence, and job satisfaction. To date, no study has been conducted to explore the moderating role of immigrant duration in the relationship between these variables. By filling those research gaps, this study may broaden the understanding of the role of language in foreign-born nurses’ job satisfaction while also being beneficial in developing strategies to reduce discrimination and retain foreign-born nurses.

**Fig. 1:**
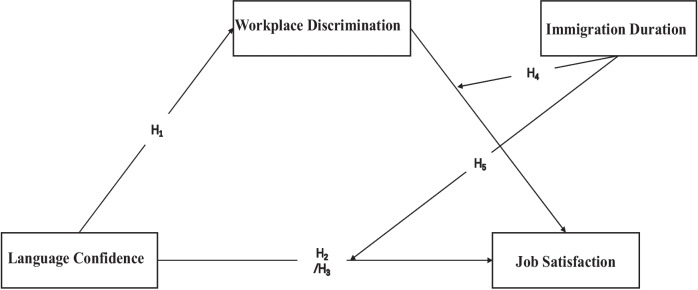
Hypothesis model of this study

This study builds on the JD–R model of job satisfaction, aimed to explore the relationship between language confidence and job satisfaction, examine the mediating role of workplace discrimination and the moderating role of immigration duration among foreign-born nurses in Japan (Fig. 1). This study propose that 1) foreign-born nurses with higher confidence in their second language perceive less workplace discrimination (*hypotheses 1*) and higher job satisfaction (*hypotheses 2*); 2) workplace discrimination mediates the relationship between language confidence and job satisfaction (*hypotheses 3*); 3) immigration duration negatively moderates the relationship between workplace discrimination and job satisfaction (*hypotheses 4*); and 4) immigration duration positively moderates the relationship between language confidence and job satisfaction (*hypotheses 5*).

## Methods

### Design

This study used a cross-sectional design. Although cross-sectional design limits the ability to establish the causal relationships among variables, it meets the need of this study to estimate the level of behavior in a population during the same time period from various places [24]. Moreover, the Strengthening the Reporting of Observational Studies in Epidemiology (STROBE) checklist was used as a report guideline (supplementary file).

### Participants

The inclusion criteria were as follows: 1) being born outside of Japan, 2) possessing a Japanese registered nurse license, and 3) being employed as a nurse at the time of the investigation. The minimum sample size was calculated to be 157 using G* Power 3.1. This calculation was based on our previous study [19] that explored the relationship between language satisfaction and job satisfaction of foreign-educated nurses in Japan using linear regression, with an effect size of 0.15, the α level of 0.05, a power of 0.90, and 12 predictors in the current study. Since traveling abroad and attending a nursing school in that country was also reported as the main method for nurse migration [1], we included both foreign-born nurses who received basic nursing education in their original country and in Japan in our current research.

### Data collection

Between June and August 2022, an online questionnaire (Google Forms, available in Japanese and English) was distributed to participants via email by the two organizations. One of these organizations is actively engaged in the education and recruitment of non-native nurses, whereas the other focuses on enlisting Chinese nurses from Japan. Additionally, questionnaires were distributed via SNS messages (WeChat and Facebook) using snowball sampling [19].

### Measurement

#### Demographic characteristics

Demographic characteristics were designed based on a previous review [25], including age, gender, marital status, nationality, place of receiving basic nursing education, nursing experience in original country, holding Japanese permanent residence, education level, work setting, immigration duration (years since migration to Japan), and annual income.

#### Workplace discrimination

Workplace discrimination refers to being treated unfairly in employment based on one or more personal characteristics such as nationality and religion [26]. Based on a previous study [27], workplace discrimination was measured using a single-item question asking participants whether they perceived themselves as having experienced discrimination at their workplace during the past 12 months. The question was scored on a five-point Likert-scale from 1 (rarely or never) to 5 (very often or continuously). An additional multiple-choice question asked if the participants had experienced discrimination in their workplace by 1) superiors or managers, 2) colleagues, 3) patients, or 4) patients' families.

#### Language confidence

Language confidence refers to a person's self-assurance and comfort levels when using a particular language [28]. It was measured using a four-point Likert scale from 1 (unconfident) to 4 (confident) by asking foreign-born nurses how confident they were with their current Japanese level, adapted from Chiba and Nakayama [29].

#### Job satisfaction

Job satisfaction was measured using the Japanese version of the Mueller-McCloskey Satisfaction Scale (MMSS) [30], developed by Mueller and McCloskey [31]. This 31-item scale covers the following eight domains: extrinsic rewards (three items), scheduling (six items), family/work balance (three items), coworkers (two items), interaction (four items), professional opportunities (four items), praise/recognition (four items), and control/responsibility (five items). Each item was scored on a five-point Likert scale (1 = very dissatisfied, 5 = very satisfied), yielding a score of 31–155. A higher score indicates a higher level of job satisfaction. The original MMSS scale was developed and available in English, Cronbach’s alpha for the original version was 0.89 [31]. This instrument was translated into Japanese using a best practice approach of translation-back translation and examined for reliability using the 6-month test–retest method, Cronbach’s alpha for the Japanese version is 0.90 [30]. Permission to use this scale was obtained from the creator and the Japanese translator. Cronbach’s alpha of scale in current study was 0.94, indicating good reliability.

#### Ethical considerations

This study was approved by the Ethics Committee of Tokyo Medical and Dental University (IRB number: C2021-013). The participants were informed of their voluntary and anonymous participation. Completion and submission of the questionnaire were regarded as consent to participate. Participants who provided an email address after completing the questionnaire received a 500-yen Amazon gift card (worth $3) as compensation for their time. Participants’ email addresses were collected for sending gift cards using a separate Google form, and they were informed of their right to decide whether or not to provide their email address for gift card sending.

#### Statistical analysis

There were no missing values, and SPSS (version 28.0, Mac) and SPSS PROCESS Macros were used in the current study. Descriptive statistics were used to present the participants’ demographics and study variables. Since the skewness and kurtosis values of job satisfaction (min -0.50, max 1.25) were within $\pm$ 2, parametric analysis was performed. For examining the association between ordinal variables (e.g., age, language confidence) and continuous variables (job satisfaction), Spearman’s correlation test was used. Student’s t-tests and one-way analysis of variance with Bonferroni correction were used to identify confounding demographic variables that may be related to job satisfaction. Variables with significance (*p* < 0.05) in the bivariate analysis were included as covariates in the final model.

Hayes’ SPSS PROCESS Macro [32] is widely used in health science research to test regression models with mediating and/or moderating effects by adjusting for covariates. Model 4 was used to assess the relationship between language confidence, workplace discrimination, and job satisfaction as well as the mediating role of workplace discrimination. The statistical significance of the indirect mediation effect on job satisfaction was tested using the 5000-time bootstrapping method with a 95% confidence interval [32]. Model 15, with the Johnson-Neyman technique, was used to examine the moderating effect of immigration duration on the relationship between workplace discrimination and job satisfaction as well as in the relationship between language confidence and job satisfaction. The moderator interaction and simple slope at five-levels—very low (-1SD), low (-0.5SD), mean (0SD), high (0.5SD), and very high (1SD)—were shown using the InterActive tool [33].

## Results

### Participants’ characteristics

Of the 206 completed questionnaires, 187 valid data (effective response rate: 91%) were analyzed. Most participants were female (91.2%), from China (96.8%), and had received a basic nursing education in their original country (92.5%) (Table 1). The mean immigration duration in Japan was 6.89 $\pm$ 4.07 years. During the past 12 months, 79.7% of the participants (*n* = 149) reported being discriminated against in the workplace, mostly by their colleagues (*n* = 112), followed by their managers or supervisors (*n* = 71), patients (*n* = 68), and patients’ families (*n* = 41).

**Table 1:** Participants’ characteristics (*n* = 187)

Characteristics	n (%)	Job satisfaction	
		**Mean (SD)**	**t/F**
Gender			0.60^a^
Female	172 (91.2)	94.61 (16.39)	
Male	15 (8.8)	91.53 (31.11)	
Marital status			-2.38^a*^
Single	73 (39.0)	91.90 (18.89)	
Married	114 (61.0)	98.21 (15.61)	
Nationality			-2.71^a**^
China	181 (96.8)	93.78 (17.60)	
Others^1^	6 (3.2)	113.50 (18.01)	
Place of receiving basic nursing education			-0.54^a^
Original country	173 (92.5)	94.02 (17.80)	
Japan	14 (7.5)	98.50 (19.41)	
Nursing experience in original country			0.11^b^
No	136 (72.7)	93.21 (18.82)	
Yes, 2-year less	32 (17.1)	94.50 (16.21)	
Yes, 2-year more	19 (10.2)	102.42 (11.16)	
Japanese permanent residence			-1.25^a^
No	163 (87.2)	93.74 (18.47)	
Yes	24 (12.8)	98.63 (12.93)	
Highest education level			0.24^b^
Vocational/Junior collage	49 (26.2)	93.42 (17.44)	
Bachelor’s degree	129 (69.0)	94.47 (18.22)	
Master’s degree and above	9 (5.8)	97.89 (17.30)	
Work setting			0.76^b^
Hospital	156 (83.4)	94.80 (17.98)	
Care institute	15 (8.0)	90.27 (16.34)	
Clinic	12 (6.4)	97.00 (17.34)	
Others^2^	4 (2.2)	84.75 (23.89)	
Annual income (included tax)			0.20^b^
Less than 3 million yen	21 (11.2)	95.04 (18.77)	
3 million ~ less than 5 million yen	118 (63.1)	93.77 (16.97)	
5 million yen ~	41 (21.9)	94.98 (19.61)	
Prefer not to answer	7 (3.8)	98.71 (23.51)	

^a^Student t-test; ^b^One way analysis of variance with Bonferroni correction; *SD *Standard deviation

^1^Others included: Mongolia (*n* = 4); Indonesia (*n* = 1); Taiwan (*n* = 1)

^2^Others included: Home-visit nursing center (*n* = 3); medical call center (*n* = 1)

*p < 0.05; **p < 0.01

### Preliminary correlation analyses and covariate examination

Married nurses reported higher job satisfaction than single nurses (t = -2.38, *p* = 0.018), and nurses who came from China reported significantly lower job satisfaction than those who did not (t = -2.71, *p* = 0.007; Table 1). Language confidence ($\rho$ = 0.200, *p* = 0.006; Table 2) was positively correlated with job satisfaction. Workplace discrimination was negatively correlated with job satisfaction ($\rho$ = -0.425, *p* < 0.001). Language confidence ($\rho$ = -0.189, *p* = 0.010) was negatively correlated with workplace discrimination. Marital status and nationality were treated as covariates in all the models.

**Table 2:** Spearman’s correlations test among job satisfaction, workplace discrimination, language confidence, age, immigration duration

Variables	Mean (SD)	1	2	3	4
1.Job satisfaction	94.36 (17.90)				
2.Age	30.01 (4.46)	0.120			
3.Immigration duration	6.89 (4.07)	0.121	0.835^***^		
4.Workplace discrimination	2.20 (0.89)	-0.425^***^	-0.115	-0.098	
5.Language confidence	2.71 (0.64)	0.200^**^	0.144^*^	0.203^**^	-0.189^**^

*SD* Standard deviation

^*^ *p* < 0.05; **p < 0.01; ***p < 0.001

### Direct and indirect effects among language confidence, workplace discrimination, and job satisfaction

After adjusting for covariates, language confidence was a negative predictor of workplace discrimination (*β* = -0.16, B = -0.22, *p* = 0.027) and a positive predictor of job satisfaction (*β* = 0.15, B = 4.02, *p* = 0.032; Table 3). Workplace discrimination was negatively predicting job satisfaction (*β* = -0.38, B = -7.55, *p* < 0.001). Hence, *hypotheses 1* and* 2* are supported. Language confidence had an indirect effect on job satisfaction through workplace discrimination (*β* = 0.06, B = 1.69, *p* < 0.05), which was 30% of the total effect. Thus, *hypothesis 3* is supported.

**Table 3:** Result of mediation effect and moderated mediation effect

Predictor	Workplace discrimination				Job satisfaction				H
	${\varvec{\beta}}$	${\varvec{B}}$	**SE**	**LLCI—ULCI**	${\varvec{\beta}}$	${\varvec{B}}$	**SE**	**LLCI—ULCI**	
**Result from mediation model (Model 4 with 5000 times bootstraps)**									
Language confidence	-0.16^*^	-0.22	0.10	-0.42 — -0.03	0.15^*^	4.02	1.86	0.34 — 7.70	H1/H2
Workplace discrimination					-0.38^***^	-7.55	1.35	-10.22 — -4.88	
Nationality	-0.13	-0.63	0.36	-1.34 — 0.08	0.12	12.72	6.65	-0.39 — 25.83	
Marital Status	-0.10	-0.17	0.13	-0.43 — 0.87	0.10	3.40	2.42	-1.38 — 8.18	
R^2^	0.06				0.24				
F	4.00^*^				14.07^***^				
**Effect path**					${\varvec{\beta}}$	${\varvec{B}}$	**SE**	**LLCI—ULCI**	
Total	Language confidence → Job satisfaction				0.21^**^	5.71	1.99	1.79 — 9.63	
Direct	Language confidence → Job satisfaction				0.15^*^	4.02	1.86	0.34 — 7.70	
Indirect	Language confidence → Workplace discrimination → Job satisfaction				0.06^*^	1.69	0.72	0.35 — 3.20	H3
**Result from moderated mediation model (Model 15 with 5000 times bootstraps)**									
Language confidence	-0.16^*^	-0.22	0.10	-0.42 — -0.03	0.13	3.53	1.86	-0.14 — 7.20	
Workplace discrimination					-0.37^***^	-7.32	1.35	-9.98 — -4.65	
Immigration duration					-0.03	-0.01	0.33	-0.67 — 0.64	
Workplace discrimination x Immigration duration					0.10	0.48	0.42	-0.36 — 1.31	H4
Language confidence x Immigration duration					0.19^**^	1.26	0.43	0.42 — 2.10	H5
Nationality	-0.13	-0.63	0.36	-1.34 — 0.07	0.11	11.76	6.53	-1.29 — 24.48	
Marital Status	-0.10	-0.17	0.13	-0.43 — 0.09	0.07	2.76	2.76	-2.29 — 7.81	
R^2^	0.06				0.28				
F	4.00^**^				9.82^***^				

*Abbreviation*: *SE* Standard Error, *LLCI* Lower-Level Confidence Interval, *ULCI* Upper-Level Confidence Interval, *H* Hypothesis

^*^ *p* < 0.05; **p < 0.01; ***p < 0.001

### Moderating role of immigration duration

In model 15, workplace discrimination significantly predicted lower job satisfaction (*β* = -0.37, B = -7.32, *p* < 0.001), whereas language confidence did not (*β* = 0.13, B = 3.53, *p* = 0.059; Table 3). The interaction between workplace discrimination and immigration duration on job satisfaction was not significant (*β* = 0.10, B = 0.48, *p* = 0.262), whereas that between language confidence and immigration duration on job satisfaction was significant (*β* = 0.19, B = 1.26, *p* = 0.004), thus rejecting *hypothesis 4* and supporting *hypothesis 5.*

**Fig. 2:**
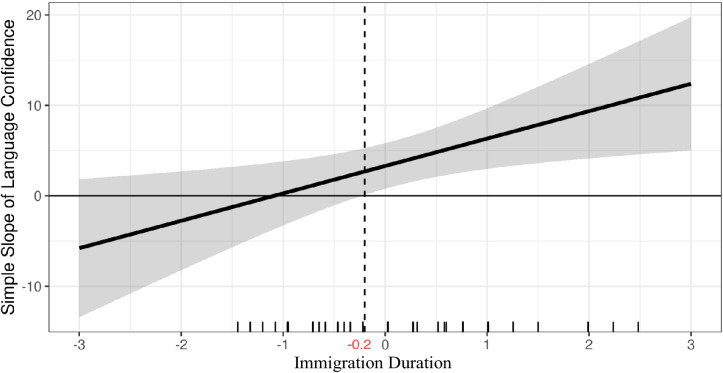
Ninety-five percent Marginal effects plot (Immigration Duration as moderator). *Note: This figure illustrates the relation between language confidence and job satisfaction across a multiple level of moderator (immigration duration). The effect of language confidence on job satisfaction become statistically significant from the right of the dotted line (-0.2)

**Fig. 3:**
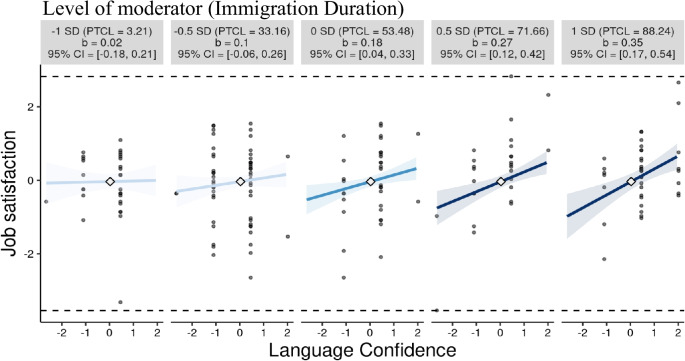
Five-level simple slope (immigration duration as moderator). Note: this figure illustrates the simple slope in a small multiple at -1 SD, -0.5 SD, 0 SD (mean), + 0.5 SD, + 1 SD levels of the moderator (immigration duration). The shaded areas indicated the 95% Confidence Interval (CI). The gray circle indicates observed data. Computed 95% confidence regions, observed data, indicators of the maximum and minimum values of the outcome (job satisfaction), and x-axes representing the full range of the focal predictor (language confidence) are provided in each graphic. All variables used in interactions were standardized (z-scores); PTCL: percentile

The Johnson-Neyman technique indicated that the effect of language confidence on job satisfaction became significant above from -0.2 standard deviations (SD) and included 46.52% of observations in immigration duration (Fig. 2). The small multiples graphic (Fig. 3) also illustrates that the effect of language confidence on job satisfaction is not significantly different from -0.5 SD of immigration duration or lower, but significant and positive at mean levels (0 SD) or higher.

## Discussion

This study indicated that nurses with higher language confidence perceived lower workplace discrimination and higher job satisfaction, whereas workplace discrimination partly mediated the relationship between language confidence and job satisfaction. This was the first study to report that workplace discrimination was a mediator in the relationship between language confidence and job satisfaction, and as immigration duration increased, this relationship strengthened among foreign-born nurses in Japan.

This study found that foreign-born nurses with lower confidence in their Japanese proficiency perceived greater workplace discrimination and lower job satisfaction. The finding of the negative relationship between language confidence and workplace discrimination was supported by Garner et al.'s qualitative study [34], indicating that non-native nurses felt discriminated against and isolated when their language proficiency was doubted in the workplace, such as being excluded from important meetings or being refused care by patients. Thus, nurses who lack confidence in their language abilities may be perceived as less competent or capable by their colleagues or superiors, potentially leading to unfair treatment or discrimination in terms of job assignments and promotional opportunities.

The positive relationship between language confidence and job satisfaction was consistent with our previous study, which indicated that foreign-educated nurses with higher satisfaction with their language proficiency had higher job satisfaction [19]. According to Zhong et al. [35], non-native nurses who are more confident in their language abilities tend to be more engaged in the workplace and actively participate in discussions. They may appear to have comfortable and effective communication in the workplace [12], facilitating better relationships with local colleagues and contributing to a more fulfilling work experience [11]. Moreover, being unfamiliar with the receiving countries’ cultural and communication styles could decrease non-native nurses’ language confidence, especially if pre-established communication styles and cultural habits seem to be inappropriate in the receiving country [36], which might decrease their job satisfaction. The mediating role of workplace discrimination in the relationship between language confidence and job satisfaction could also be explained by the fact that increased language confidence reduces language issues, leading to effective communication and the mitigation of discrimination in the workplace, which in turn enhances overall job satisfaction [11]. A previous study also suggested that decreasing language discrimination and racism in the workplace could contribute to improving foreign-educated nurses’ well-being and job satisfaction, as well as preventing the threat to patients’ safety [9]. Thus, these results underscore the importance of language support and cultural education among foreign-born nurses as potential strategies to decrease workplace discrimination and improve their satisfaction.

The positive moderating effect of immigration duration in the current study indicated that as immigration duration increased, the influence of language confidence on job satisfaction increased. According to the Johnson-Neyman point method, nurses who had lived relatively long in Japan and perceived higher confidence in their language proficiency reported significantly higher job satisfaction; however, this relationship became less significant or non-significant for nurses with lower immigration length. In the early stages of immigration, foreign-born nurses, regardless of whether they have high or low language confidence, may primarily concentrate on adjusting to a new environment, experiencing culture shock, and understanding the basics of their new surroundings [8]. These challenges may reduce the impact of language proficiency on job satisfaction. In contrast, nurses who have lived in the receiving country for a longer period are more likely to adapt and integrate into the culture and work lifestyle of that country [17]. Furthermore, compared to the early stages of immigration, the benefits of having strong language proficiency might become more apparent, such as increasing the opportunity to access specialized and graduate nursing programs, which could contribute to career development and boost overall job satisfaction in the receiving countries.

### Implications for nursing and health policy

#### Foreign-born nurses with higher confidence in their second language perceive less workplace discrimination and higher job satisfaction

As Japan has been identified as a homogenous society, it is challenging to accept individuals from different cultural backgrounds [37]. Increasing the interaction opportunities between local workers and foreign-born nurses would foster mutual understanding and a sense of cultural differences, contributing to promoting collaboration and a decrease in stereotype discrimination. The implementation of mentorship programs, which pair non-native nurses with experienced native nurses, could potentially improve this interaction. These mentors can provide crucial guidance and support to encourage and help them gain confidence in practical language use and understand local cultural norms and communication styles, contributing to enhanced job satisfaction.

#### Workplace discrimination mediates the relationship between language confidence and job satisfaction

The government should establish regulations and laws to ensure fair and non-discriminatory hiring practices by employers and institutions. The government should provide funding and resources for healthcare facilities to assist in developing and implementing training programs (e.g., language, local culture) for non-native nurses and lectures regarding unconscious workplace discrimination for local workers. Organizational managers and policymakers should re-evaluate the work environment, establish policies and strategies to create an inclusive work environment, and actively combat discriminatory treatment to mitigate the negative impact of discrimination on non-native nurses’ job satisfaction.

#### Immigration duration positively moderates the relationship between language confidence and job satisfaction

Tailored training and support for foreign-born nurses with different immigration lengths are recommended, such as language and receiving countries’ cultural training for newly arrived nurses, cross-cultural communication skills training, and career advancement support for nurses who lived longer and are relatively familiar with the receiving country.

### Limitations and future research

This study has several limitations. The cross-sectional study design limited the causal relationship between language confidence and job satisfaction. Therefore, future studies with longitudinal designs are required. As more than 95% of the participants were Chinese, the results might be difficult to generalize to nurses of other nationalities in Japan. Future studies on foreign-born nurses of various nationalities in Japan are required to address this issue.

Workplace discrimination and language confidence were measured using single-item scales. Future studies utilizing multi-item measurements of workplace discrimination and language confidence should explore foreign-born nurses’ perspectives in depth. The current study only offers numeric data; future qualitative or mixed-method studies on the nurses’ perspective of workplace discrimination, language, and job satisfaction would be useful to verify the result of this study and gain a better understanding of the relationship between these variables. Moreover, only 6.0% of the variance in workplace discrimination was explored in this study, indicating that future studies should explore more specific predictors of workplace discrimination to gain a deeper understanding.

This study used an online self-administered questionnaire, which may have retained reporting bias. Future studies with more objective data, such as language proficiency test results and evaluation of language proficiency by linguistic experts, would provide more accurate data. Participants were recruited through an online questionnaire; it is likely that nurses who were more comfortable with online environments responded to the survey. Future studies could implement both paper-based and online questionnaires to recruit more participants and address this issue.

## Conclusion

This study showed that foreign-born nurses with higher confidence in their Japanese language proficiency were more satisfied with their jobs in Japan. Nurses with lower language confidence were more likely to experience workplace discrimination, resulting in decreased job satisfaction. The relationship between language confidence and job satisfaction was significantly enhanced with longer years of migration. To maintain and improve foreign-born nurses’ job satisfaction in Japan, organizations and nurse managers should establish an equal and supportive work environment and provide tailored training and support.

### Supplementary Information

Supplementary Material 1.

## Data Availability

The datasets used and/or analyzed in the current study can be made available by the corresponding author upon reasonable request.

## References

[1] Socha-Dietrich K, Dumont JC (2021). International migration and movement of nursing personnel to and within OECD countries - 2000 to 2018: developments in countries of destination and impact on countries of origin. OECD Health Working Paper.

[2] Buchan J, Catton H, Shaffer FA (2022). Sustain and retain in 2022 and beyond: the global nursing workforce and the Covid-19 pandemic. Int Counc Nurs.

[3] Khan T, Abimbola S, Kyobutungi C, Pai M (2022). How we classify countries and people—and why it matters. BMJ Glob Health.

[4] Marć M, Bartosiewicz A, Burzyńska J, Chmiel Z, Januszewicz P (2019). A nursing shortage – a prospect of global and local policies. Int Nurs Rev.

[5] Japanese Immigration Services Agency. Statistics on foreign residents in Japan. 2023. Available at: https://www.moj.go.jp/isa/policies/statistics/toukei_ichiran_touroku.html. Accessed 22 Dec 2023.

[6] Japanese Ministry of Health, Labour and Welfare. Analysis of foreign job applicants. 2023. Available at: https://www.mhlw.go.jp/content/11601000/000840138.pdf. Accessed 22 Dec 2023.

[7] Villamin P, Lopez V, Thapa DK, Cleary M. Retention and turnover among migrant nurses: a scoping review. Int Nurs Rev. 2023:1–15. 10.1111/inr.12861. 10.1111/inr.12861 37467162

[8] Balante J, van den Broek D, White K (2021). How does culture influence work experience in a foreign country? An umbrella review of the cultural challenges faced by internationally educated nurses. Int J Nurs Stud.

[9] Viken B, Solum EM, Lyberg A (2018). Foreign educated nurses’ work experiences and patient safety—a systematic review of qualitative studies. Nurs Open.

[10] Kurniati A, Chen CM, Efendi F, Ogawa R (2017). A deskilling and challenging journey: the lived experience of Indonesian nurse returnees. Int Nurs Rev.

[11] Pressley C, Newton D, Garside J, Simkhada P, Simkhada B (2022). Global migration and factors that support acculturation and retention of international nurses: a systematic review. Int J Nurs Stud Adv.

[12] Hammer K. Sociocultural integration and second language proficiency following migration. In: The linguistic integration of adult migrants. 2017. p. 2–7. 10.1515/9783110477498-012.

[13] Bakker AB, Demerouti E (2017). Job demands–resources theory: taking stock and looking forward. J Occup Health Psychol.

[14] Hassard J, Wang W, Delic L, Grudyte I, Dale-Hewitt V, Thomson L (2023). Pregnancy-related discrimination and expectant workers' psychological well-being and work engagement: understanding the moderating role of job resources. Int J Workplace Health Manag.

[15] Yeung DY, Zhou X, Chong S (2021). Perceived age discrimination in the workplace: the mediating roles of job resources and demands. J Manag Psychol.

[16] Madera JM, Dawson M, Neal JA (2014). Managing language barriers in the workplace: the roles of job demands and resources on turnover intentions. Int J Hosp Manag.

[17] Primeau MD, St-Pierre I, Ortmann J, Kilpatrick K, Covell CL (2021). Correlates of career satisfaction in internationally educated nurses: a cross-sectional survey-based study. Int J Nurs Stud.

[18] Zanjani ME, Ziaian T, Ullrich S, Fooladi E (2021). Overseas qualified nurses’ sociocultural adaptation into the Australian healthcare system: a cross-sectional study. Collegian.

[19] Hua J, Kondo A, Wang C, Ganchuluun S (2023). Job satisfaction, intention to leave, and related factors among foreign-educated nurses in Japan: a cross-sectional study. J Nurs Manag.

[20] Bakker AB, Demerouti E (2007). The job demands-resources model: state of the art. J Manag Psychol.

[21] Elizabeth M, Richard WB, Angelina RS (2022). Perceived ethnic discrimination and cognitive function: a 12-year longitudinal study of Mexican-origin adults. Soc Sci Med.

[22] Scholaske L, Wadhwa PD, Entringer S (2021). Acculturation and biological stress markers: a systematic review. Psychoneuroendocrinology.

[23] Deng XQ, Shi BG (2013). Migrant children’s perceived discrmination and self-esteem: the effect of social support and migrant duration. Chin J Spec Educ.

[24] Cohen L, Manion L, Morrison K. Research methods in education: survey, longitudinal, cross-sectional and trend studies. 8th ed. London: Routledge; 2017.

[25] Wang Z, Jing X (2018). Job satisfaction among immigrant workers: a review of determinants. Soc Indic Res.

[26] Colella A, King E (2018). The Oxford handbook of workplace discrimination.

[27] Wesołowska K, Elovainio M, Komulainen K, Hietapakka L, Heponiemi T (2020). Nativity status and workplace discrimination in registered nurses: testing the mediating role of psychosocial work characteristics. J Adv Nurs.

[28] Noels KA, Pon G, Clément R (1996). Language, identity, and adjustment: the role of linguistic self-confidence in the acculturation process. J Lang Soc Psychol.

[29] Chiba Y, Nakayama T (2016). Cultural immersion through international experiences among Japanese nurses: present status, future intentions, and perceived barriers. Jpn J Nurs Sci.

[30] Shijiki Y (1997). Validity and reliablity of the Japanese McCloskey and Mueller Satisfaction Scale (JMMSS). Bulletin of Tokyo Metropolitan College of Medical Technology.

[31] Mueller CW, McCloskey JC (1990). Nurses’ job satisfaction: a proposed measure. Nurs Res.

[32] Hayes AF (2021). Introduction to mediation, moderation, and conditional process analysis: a regression-based approach.

[33] McCabe CJ, Kim DS, King KM (2018). Improving present practices in the visual display of interactions. Adv Methods Pract Psychol Sci.

[34] Garner SL, Conroy SF, Bader SG (2015). Nurse migration from India: a literature review. Int J Nurs Stud.

[35] Zhong Y, McKenna L, Copnell B (2017). What are Chinese nurses’ experiences whilst working overseas? A narrative scoping review. Int J Nurs Stud.

[36] Bond S, Clair M, Helen W (2020). The experiences of international nurses and midwives transitioning to work in the UK: a qualitative synthesis of the literature from 2010 to 2019. Int J Nurs Stud.

[37] Demelius Y (2020). Multiculturalism in a “homogeneous” society from the perspectives of an intercultural event in Japan. Asian Anthropol.

